# Using Amino Acid Physicochemical Distance Transformation for Fast Protein Remote Homology Detection

**DOI:** 10.1371/journal.pone.0046633

**Published:** 2012-09-28

**Authors:** Bin Liu, Xiaolong Wang, Qingcai Chen, Qiwen Dong, Xun Lan

**Affiliations:** 1 School of Computer Science and Technology, Harbin Institute of Technology Shenzhen Graduate School, Shenzhen, Guangdong, People's Republic of China; 2 Key Laboratory of Network Oriented Intelligent Computation, Harbin Institute of Technology Shenzhen Graduate School, Shenzhen, Guangdong, People's Republic of China; 3 School of Computer Science, Fudan University, Shanghai, People's Republic of China; 4 Department of Biomedical Informatics, The Ohio State University, Columbus, Ohio, United States of America; University of Michigan, United States of America

## Abstract

Protein remote homology detection is one of the most important problems in bioinformatics. Discriminative methods such as support vector machines (SVM) have shown superior performance. However, the performance of SVM-based methods depends on the vector representations of the protein sequences. Prior works have demonstrated that sequence-order effects are relevant for discrimination, but little work has explored how to incorporate the sequence-order information along with the amino acid physicochemical properties into the prediction. In order to incorporate the sequence-order effects into the protein remote homology detection, the physicochemical distance transformation (PDT) method is proposed. Each protein sequence is converted into a series of numbers by using the physicochemical property scores in the amino acid index (AAIndex), and then the sequence is converted into a fixed length vector by PDT. The sequence-order information can be efficiently included into the feature vector with little computational cost by this approach. Finally, the feature vectors are input into a support vector machine classifier to detect the protein remote homologies. Our experiments on a well-known benchmark show the proposed method SVM-PDT achieves superior or comparable performance with current state-of-the-art methods and its computational cost is considerably superior to those of other methods. When the evolutionary information extracted from the frequency profiles is combined with the PDT method, the profile-based PDT approach can improve the performance by 3.4% and 11.4% in terms of ROC score and ROC50 score respectively. The local sequence-order information of the protein can be efficiently captured by the proposed PDT and the physicochemical properties extracted from the amino acid index are incorporated into the prediction. The physicochemical distance transformation provides a general framework, which would be a valuable tool for protein-level study.

## Introduction

A vast amount of protein sequences has been obtained with the development of large-scale sequencing techniques, which need to be classified into structural and functional classes by means of homologies. Therefore, fast and accurate algorithms that can automatically detect the protein remote homologies are needed. However, protein remote homology detection, referring to the detection of evolutional homology in proteins with low similarities, is still a challenging problem in bioinformatics.

Because of the importance of remote homology detection, it has been intensively studied for a decade. Many computational methods have been proposed to address this problem, which can be split into three groups: pairwise comparison methods, generative models and discriminative algorithms. Pairwise comparison methods measure the pairwise similarities between protein sequences. For example, in the pairwise method [1], each protein sequence is represented as a vector of pairwise similarities to all protein sequences in the training set. Smith-Waterman dynamic programming algorithm [2] is adopted to calculate an optimal score for similarity according to a predefined objective function. RANKPROP [3] depends on a precomputed network of pairwise protein similarities. LESTAT [4] detects remote homologies by constructing the iterative profiles. Generative models induce a probability distribution over the protein family and try to generate the unknown proteins as new member of the family from the stochastic model. For example, hidden Markov model (HMM) [5] can be trained iteratively in a semi-supervised manner, which uses both positively labeled and unlabeled samples of a particular family by pulling in close homology and adding them to the positive set [6]. Recent methods have applied the discriminative algorithms for accurate remote homology detection. Different from the generative methods, the discriminative methods lean a combination of the features that can discriminate the protein families. Among these methods, the top-performing methods use the support vector machines (SVM) [7] to build the discriminative framework. The core component in the SVM is the calculation of the kernel functions, which measure the difference between any two pair of samples. Many approaches have been proposed to build the kernel functions. LA kernel [8] measures the similarity between a pair of proteins by taking all the optimal local alignment scores with gaps between all possible subsequences into account. SVM-HUSTLE [9] builds a SVM classifier for a query sequence by training on a collection of representative high-confidence training sets, recruits additional sequences. Lingner and Meinicke present a kernel based on the average word similarity between two protein sequences [10]. Other kernels are built by using the sequence features, such as the motifs [11], [12], [13], mismatch [14], SVM-I-sites [15], SVM-n-peptide [16], N-gram [17], Patterns [18], SVM-BALSA [19] and so on. The advantage of these methods is they don't need computational expensive feature generation step, but their Receiver Operating Characteristic (ROC) scores generally is low, ranging from 0.87 to 0.90 on the standard SCOP 1.53 benchmark. Our prior work shows that the performance of these methods can be improved by using the latent semantic analysis (LSA) [20]. Some top-performing methods employ the evolutional information extracted from the profiles. These methods need an additional alignment step to generate the profiles by searching against a non-redundant database, which leads to higher computational cost. SW-PSSM [21] employs the profile-to-profile scoring schemes for measuring the similarity between pairs of proteins. Profile kernel [22] extracts the short substrings according to the profile-based ungapped alignment scores. Top-*n*-grams [23] extract the profile-based patterns by considering the most frequent elements in the profiles. The feature vector of ILP-SVM [24] is based on the frequent patterns in the profiles detected by inductive logic programming. The recently proposed ACC method [25] treats the protein sequence as a time sequence and applies the auto-cross covariance (ACC) transformation to capture the correlation between any two properties in the profiles. The ROC scores of these methods generally range from 0.92–0.98. However, due to the high computational cost in the feature generation stage, applying these profile-based methods to large-scale remote homology detection is often unfeasible. Other profile-based methods focus on the development of more sensitive profiles, such as HHsearch method [26] proposes a novel profile based on hidden Markov models. FFAS [27] is another profile-profile alignment method, which is based on a new procedure for profile generation that takes into account all the relations within the family. COMPASS [28] generates numerical profiles, constructs optimal profile-profile alignments and estimates the statistical significance of the corresponding alignment scores. Some web servers implementing the profile-profile alignment algorithms are available, including COMA[29], PHYRE[30], GenThreader[31], FORTE [32] and webPRC [33].

A key step to improve the performance of the SVM-based methods is to find a fast and accurate representation of protein sequence. Although the profile-based features improve the accuracy by considering the evolutional information extracted from the profiles, the high computational cost prevents the widespread application of these methods to a large database. By contrast, the methods based on the sequence composition information can generate the feature vectors with low computational cost. However, if a protein is represented by its amino acid composition alone, all the information of its sequence order and sequence length is totally lost. Previous studies show that the sequence-order effects are relevant for remote homology detection. Lingner and Meinicke propose a method based on distances between short oligomers [34], which outperforms other position independent approaches. In ACC method, the sequence-order is captured by the autocross-covariance (ACC) transformation[35]. SVM-HMMSTR [36] can capture the sequential ordering of the local structures. SVM-RQA [37] uses the recurrence quantification analysis (RQA) to detect the autocorrelation patterns along the protein sequences. Previous studies show that the predictive performance of other problems can also be improved by adding the sequence order information into the feature vectors, such as membrane protein family prediction[38], structural class prediction [39], [40], and secondary structure content prediction [41], [42]. The difficulty to include the sequence-order information into the prediction is that protein sequence lengths vary widely. Besides, the protein sequence information only describes the amino acid composition of a protein without considering the physicochemical properties of the amino acids. Because protein structure and function are more conserved during evolutionary process, the similarity between two distantly related proteins may lie in the physicochemical properties of the amino acids rather than the sequence identities. In this study, we propose the physicochemical distance transformation (PDT) method for remote homology detection, which is able to include the local sequence-order information of the entire protein sequences and use the amino acid physicochemical properties in the Amino Acid Index (AAIndex) [43]. The protein sequences are converted into fixed length vectors by using PDT, and then input to a SVM classifier for the prediction. Testing on the SCOP 1.53 benchmark, we show that the proposed method (SVM-PDT) performs similar or better than other SVM-based methods with a significant decrease in computational time. Finally, the performance of SVM-PDT is further improved by taking the evolutionary information extracted from the frequency profiles into account.

## Results

### Comparative results of the methods based on sequence composition information

In order to compare the proposed sequence-based PDT vectorization approach with other relevant protein remote homology detection methods, the proposed method SVM-PDT was evaluated on the widely used SCOP 1.53 dataset to give an unbiased comparison with prior methods that are based on sequence composition information.

**Figure 1:**
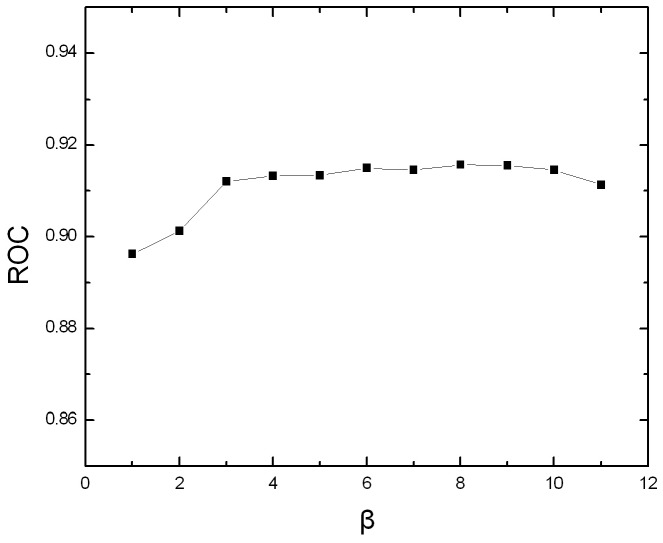
The average ROC scores of the sequence-based PDT approach with different *β* values on SCOP 1.53 dataset.

The value of *β* would impact the performance of SVM-PDT (see method section for more information). *β* can be any integer between 1 and *L*-1, where *L* is the shortest protein sequence in the dataset. The average ROC scores obtained by using different *β* values are shown in Figure 1. As we can see from the figure, the performance increases dramatically when *β* is less than 4, and then turns stable, indicating longer distances between two amino acids along the protein sequences are more important for the discrimination. The optimal value of *β* is selected as 8 in this study.

Although previous study tuned both the features and SVM parameters for each protein family, in order to evaluate the robustness and generalization of the proposed PDT vectorization approach, no feature selection was performed to select the best features for the proteins or the families. All the 531 amino acid indices were used for predicting each family. However, in order to give an unbiased comparison with another AAIndex-based method SVM-PCD [44], we followed their approach to select the most appropriate kernel function for each family. Both the quadratic and RBF kernels were evaluated. Finally, 26 families used quadratic kernel and 28 families used RBF kernel. The results of SVM-PDT and other approaches are summarized in Table 1. The results indicate the PDT approach is well-comparable with other state-of-the-art methods. Although the local alignment kernel method SVM-LA shows better average ROC and ROC50 scores, the performance of this method depends on several hyperparameters and its computational cost is much higher than our approach (see computational efficiency section for more information). By contrast, our method only has one parameter *β*, and doesn't need any time consuming local alignment step. SVM-PDT outperforms other feature-space based methods as well as the Pairwise and the WCM kernels. Therefore, the proposed sequence-based PDT approach is one of the best methods which don't need any time consuming local or multiple alignment.

**Table 1:** Comparison against the methods based on sequence composition information.

Average ROC and ROC50 scores			
Methods	ROC	ROC50	Source
SVM-PDT (*β* = 8)	0.916	0.626	This study
SVM-RQA	0.912	0.441	[37]
SVM-PCD	0.906	NA	[44]
SVM-WCM	0.904	0.445	[10]
SVM-LA(*β* = 0.5)	0.925	0.649	[8]
Mismatch	0.872	0.400	[14]
SVM-Pairwise	0.901	0.399	[1]
ODH Monomer	0.914	0.455	[10]

NA refers to the unreported result.

### Comparison with closely related methods

Beside our method, several other methods attempted to predict protein remote homologies based on AAIndex [43]. Both SVM-PCD [44] and SVM-RQA [37] take the physicochemical information extracted from the AAIndex into consideration. SVM-PCD is based on the normalized distribution of the average AAIndex value over all sequential 4-mers in a protein sequence. SVM-RQA uses both the AAIndex and the recurrence quantification analysis (RQA) to detect the auto correlation patterns along a protein sequence. Our method outperforms both of the two methods. The key difference among the three methods is that our method takes the average effects of any two amino acids within a given short distance (8 in this study) in a protein sequence into consideration by using the proposed PDT vectorization approach, while the other two methods only use the local sequence information, for example SVM-PCD only uses the 4-mer local information. We conclude that the sequence-order information is relevant for discrimination. Previous study also demonstrated the importance of the sequence-order information. Lingner and Meinicke proposed the ODH Monomer method [34], which is based on the distances between short oligomers. Although this method considers the distances for all possible pair of K-mers, only the amino acid composition of the sequence is not enough to accurately detect the remote homologies. By contrast, our approach makes use of both the physicochemical properties of amino acids extracted from the Amino Acid index and the local sequence-order information detected by using PDT vectorization approach. As can be seen from Table 1, our approach outperforms ODH Monomer, especially in terms of the average ROC50 score.

### Robustness of the physicochemical distance transformation approach

**Figure 2:**
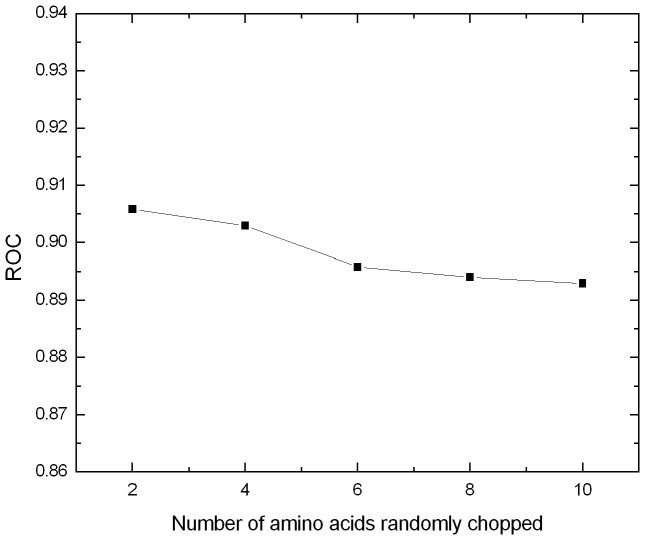
The influence of the number of randomly chopped amino acids within the beginning 20 amino acids from the N-terminus of the target proteins on the performance.

In order to investigate the robustness of the proposed physicochemical distance transformation method, a controlled experiment was conducted. In this experiment, a few amino acids within the beginning 20 amino acids from the N-terminus of the target proteins were randomly chopped. The influence of the number of randomly chopped amino acids on the performance is shown in Figure 2. The predictive performance slightly decreases (from 0.906 to 0.893 in terms of ROC) when the number of chopped amino acids increases. Chopping amino acids from the N-terminus of the proteins results in missing part of the local sequence-order information, which is the reason for the performance decrease. Because the physicochemical distance transformation approach is able to capture all the local sequence-order information (up to the distance of *β*) along a whole protein sequence, missing part of the local sequence-order information only has minor influence on the performance.

### Correlations between discriminative features and protein families

The sequence-specific weight learnt from the SVM training process can be used to calculate the discriminant weight for each feature for the interpretation of the importance of the features. Following the study [10], given the weight vector of a set of *N* sequences obtained from the kernel-based training α = [α_1_, α_2_, α_3_, …, α*_N_*], the discriminant weight vector *w* in the feature space can be calculated by the following equation:
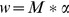
(1)


Where *M* is the matrix of sequence representatives. The magnitude of the element in *w* represents the discriminative power of the corresponding feature.

Ten most discriminative features of SVM-PDT were selected from each of the four target SCOP 1.53 families and the results are shown in Table 2. We observed a few family-specific λ variables and indices, majority of which are highly consistent with current understanding of the structure of the protein families. For example, not surprisingly, indices 130 (Surface and inside volumes in globular proteins) and 480 (Physicochemical basis of amino acid hydrophobicity scales) are discriminative features of one of these four families, family 7.3.5.2 (Spider toxins). Hydrophobicity of the protein amino acid composition is critical for the three dimensional structure of the protein. Small molecular like spider toxins may consist of more hydrophilic amino acid for the high ratio of the surface and buried amino acid. Interestingly, all the top ten most discriminative features of family 2.1.1.2 (Immunoglobin C1 set domain) have the same λ value of 2, indicating the importance of such λ value for this protein family. It indeed reflects the structure property of the C1 set domain, which is formed by beta sheets. The hydrogen bonds within the beta sheets are formed in a periodic pattern with one bond per every two amino acid in the protein sequence, supporting the correlation of amino acid with a distance 2 as a feature of this group of protein. Another example is that protein family 1.41.1.5 (calmodulin) is best described with index 453 (Averaged turn propensities in a transmembrane helix) and λ values of 3, which are associated with the helix dominated structure of this family of protein. Some indices with different λ values all show strong discriminative power for specific families, for example, the index 453 (Averaged turn propensities in a transmembrane helix) with λ values of 1, 3, 5, 6 and 7 are all among the top ten most discriminative features for family 1.41.1.2 (S100 proteins). The same pattern is also observed for other protein families listed in Table 2. Another important observation is that indices 453 (Averaged turn propensities in a transmembrane helix) and 92 (Helix initiation parameter at posision i-1) show strong discriminative power for both families 1.41.1.2 and 1.41.1.5. The two families belong to the same superfamily EF-hand, indicating the importance of these two indices for the two biologically relevant families.

**Table 2:** Ordered list of discriminative features of SVM-PDT.

Family 7.3.5.2				Family 2.1.1.2			Family 1.41.1.2			Family 1.41.1.5		
#	λ	AAIndex	Weight	λ	AAIndex	Weight	λ	AAIndex	Weight	λ	AAIndex	Weight
1	6	404	15.3	2	248	−96.6	1	453	180.6	7	453	16.7
2	6	130	15.0	2	210	−95.7	7	453	166.2	3	453	15.9
3	6	480	13.7	2	531	−94.6	3	453	162.1	3	92	13.5
4	2	480	13.0	2	147	−92.7	5	453	131.7	2	326	−12.5
5	2	404	13.0	2	516	−90.3	3	92	128.6	3	146	11.9
6	6	147	12.8	2	321	−90.0	7	92	127.6	1	453	11.8
7	1	155	12.7	2	130	−88.5	6	453	123.6	8	387	11.8
8	6	407	12.6	2	242	−88.4	5	89	120.1	2	92	−11.4
9	2	147	12.5	2	517	−87.6	5	92	118.6	2	453	−11.3
10	1	70	11.9	2	503	−87.5	1	92	112.0	1	293	11.3

List of 10 most discriminative features of four selected families for SVM-PDT. The features are sorted in descending order according to their absolute discriminative weight. For the detailed information of each index shown in this table, please refer to Text S1.

Hydrophobicity related indices are the most important discriminative features, which is consistent with previous study [37]. It is because hydrophobicity has been shown to significantly correlate with protein's structure [45]. Structure related indices are also abundant in the most discriminative features, because their distinctive periodicities in amino acid ordering could discriminative protein families. Based on the above analysis, some most discriminative indices indeed reflect the properties of the target protein families, which could explain the reason why the proposed PDT works for the protein remote homology detection.

### Incorporating evolutionary information into physicochemical distance transformation

**Figure 3:**
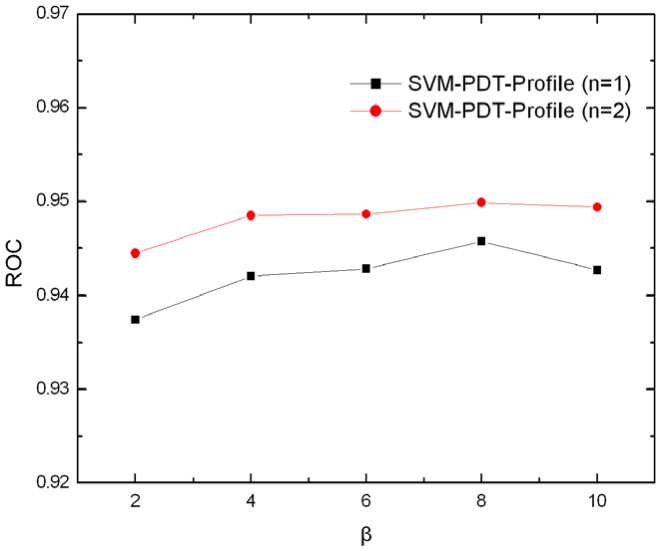
The average ROC scores of the profile-based PDT approach with different values of β.

Previous studies demonstrated that evolutionary information can improve the performance of remote homology detection. In this study, the evolutionary information imbedded in the frequency profiles is extracted and incorporated into the proposed physicochemical distance transformation approach. Each protein sequence is represented by the combination of the *n*-th most frequent amino acids in the frequency profile, and then the physicochemical distance transformation is performed on the new protein sequence to convert it into fixed length vector. In such a way, the evolutionary information is taken into the prediction. In this study, the first and second most frequent amino acids in the frequency profiles are studied, because these amino acids show better discriminative power than the others in our previous study [23]. The average ROC scores for different values of *β* are shown in Figure 3. As can be seen, the value of *β* has minor influence on the average ROC scores. The optimal value is 8 for both the profile-based PDT methods with *n* = 1 and 2. Table 3 summarizes the performance of various homology detection methods, which are all profile-based methods. As expected, compared with the sequence-based PDT method (SVM-PDT), the profile-based PDT method (SVM-PDT-Profile) can improve the performance by 3.4% and 11.4% in terms of ROC score and ROC50 score respectively, indicating the evolutionary information extracted from the frequency profiles is critical for discrimination. SVM-PDT-Profile outperforms SVM-Top-n-gram, which is another profile-based approach extracting the evolutionary information from the frequency profiles in a different way. HHearch is one of the best protein remote detection methods. It employs a novel profile based on hidden Markov models. SVM-PDT-Profile outperforms HHsearch in terms of ROC score, but HHsearch shows better ROC50 score. The main difference between HHsearch and other profile-based methods is that HHsearch employs the HMM-based profile, which is able to increase the alignment quality. ROC50 score is more sensitive to the alignment quality. This can explain the reason why HHsearch shows higher ROC50 score. For the more general performance measure ROC score, it can measure the overall performance of a method. The proposed profile-based PDT method shows the highest ROC score, reflecting combing the evolutionary information extracted from the frequency profiles and the physicochemical property scores in the amino acid index is a suitable approach to improve the performance of remote homology detection.

**Table 3:** Comparison against the profile-based methods.

Average ROC and ROC50 scores			
Methods	ROC	ROC50	Source
SVM-PDT (*β* = 8)	0.916	0.626	This study
SVM-PDT-Profile (*β* = 8, *n* = 1)	0.946	0.715	This study
SVM-PDT-Profile (*β* = 8, *n* = 2)	0.950	0.740	This study
SVM-Top-n-gram (*n* = 2)	0.923	0.713	[23]
HHSearch	0.915	0.990	*

* The results of HHsearch are obtained by in-house implementation of the hhsuite package.

### Computational efficiency

In order to detect the protein remote homology for a large-scale database, methods with low computational cost are required. Currently, the top-performing methods are mainly based on SVM. The computational cost of the vectorization step of these methods, that is to convert the proteins into fixed length vectors, is a main bottleneck preventing the widespread application of these methods to large databases, for example the SVM-LA [8] requires a time consuming local alignment step and the profile-based method SW-PSSM [21] needs to search the query sequence against a non-redundant database to get the sequence profiles for measuring the similarity between two protein sequences. By contrast, our sequence-based PDT method (SVM-PDT) and other string-kernel-based methods such as Mismatch [14] do not require any computational expensive step to generate the feature vectors, so they need less running time for the prediction. As reported by Hochreiter et al. [46], the running time of SVM-LA [8] and SW-PSSM [21] on a dataset of 20000 sequences is 550 hours and 620 hours, respectively, while Mismatch method [14] only needs 380 seconds on the same dataset, which is four orders of magnitude faster than SVM-LA and SW-PSSM.

In order to further illustrate the efficiency of the proposed method, the time complexity of the proposed sequence-based physicochemical distance transformation (PDT) is given. In this approach, each sequence-order information variable 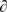 of a query sequence can be calculated by equation (5) and (6) with a time complexity of *O*(*l*), where *l* is the length of the sequence. The total number of 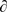 variables is *Nβ*, where *N* is the total number of the indices in the AAIndex, here the number is 531 (see method section). The optimal value of *β* is 8 as illustrated above. Therefore, the time complexity of physicochemical distance transformation is O(*Nβl*). All the 4352 protein sequences in the SCOP 1.53 dataset can be converted into fixed length vectors via using PDT with *β* value of 8 in 200 seconds. This test was performed on a personal computer with CPU of 2.8 GHz and memory of 4GB.

Sequence-based PDT method is an efficient approach for large scale dataset prediction with low computational cost. In contrast, the profile-based PDT method is suitable for small dataset and it can achieve higher accuracy. Compared with the sequence-based PDT method, the profile-based PDT approach requires an additional step to generate the frequency profiles. The running time for this step is dependent on the database size and number of samples.

## Discussion

Protein remote homology detection is to detect the structure homology in evolutionarily related protein with low sequence similarity. Discriminative methods based on support vector machine (SVM) are the most effective and accurate methods for protein remote homology detection. The performance of the SVM-based methods depends on the representation of protein sequences. The most straightforward approach to convert the sequences into fixed-length vectors is to use the amino acid composition, such as Mismatch [14], N-gram [17], motif [13] and so on. However, these methods often fail to accurately predict the proteins sharing low sequence similarity. Other methods improved the predictive performance by using the evolutional information extracted from the profiles, such as Profile [22], SW-PSSM [21], SVM-Top-N-gram [23], ACC [25]. Although these methods show the state-of-the-art performance, the profile-based methods are computational expensive preventing the application to large-scale databases. Amino acid index (AAIndex) [43] contains the physicochemical properties of the 20 standard amino acids, which is a suitable source for improving the predictive performance. Two kinds of PDT-based approaches are proposed. One is the sequence-based PDT method, which only uses the sequence information. Another one is the profile-based PDT method, which incorporates the evolutionary information extracted from the frequency profiles. Both of the two approaches extract the physicochemical properties from the AAIndex and take the local sequence-order information of the proteins into consideration. The performance of sequence-based PDT method is comparable with other methods, and its computational cost is significantly less than that of the local alignment method SVM-LA [8] and the profile-based method SW-PSSM [21]. By employing the evolutionary information, the performance of profile-based PDT method can be further improved, but as other profile-based methods it requires an additional step to generate the profiles. These two methods are complementary. The sequence-based PDT method is suitable for large scale prediction with low computational cost. The profile-based PDT method can achieve higher precision, but higher computational cost is required. The main contribution of this study is to propose two PDT-based protein representations. By measuring the correlation between any two amino acids for a given distance λ along a protein sequence (for the profile-based PDT, the protein sequence is derived from the frequency profile), the local sequence-order information can be imbedded into the final feature vector. Some other methods have used the amino acid position information in different approaches and demonstrated that it can improve the predictive performance. However, most of these methods suffer from certain shortcomings. The feature vectors of ODH Monomer [34] are derived from the distance histograms for any possible pair of *K*-mer, and this leads to a extremely large feature space, especially when multi-oligomer is used. A recently proposed method SVM-ACC [25] treats the protein sequences as time sequences. The auto-cross covariance transformation (ACC) is performed on the numerical sequences derived from the PSSM. Compared with the methods without using the sequence-order information, the performance improvement is significant. However, this profile-based method only incorporates the information extracted from the PSSMs without using any amino acid physicochemical properties. The proposed method PDT approach uses both the sequence-order information and the physicochemical properties derived from AAIndex. Another advantage of our approach arises from the explicit feature space representation: the possibility to measure the correlations between the discriminative features and protein families. By analyzing these correlations, some family-specific λ variables and indices are observed, which would be useful for the researchers who are interested in finding the characteristic features of protein families. By contrast, another AAIndex-based method SVM-RQA [37] uses the nonlinear recurrence quantification analysis (RQA) to measure the similarity between two protein sequences, which makes it difficult to explore the importance of the features for the protein families and therefore it cannot give any additional useful information. The proposed physicochemical distance transformation provides a general framework that can convert the proteins into fixed length vectors. It takes both the sequence-order information and the amino acid physicochemical properties extracted from the AAIndex into consideration. Further studies will focus on applying the PDT to other tasks in bioinformatics, such as protein-protein interaction prediction, and other protein-level classification tasks.

## Methods

### Dataset description

A common benchmark [1] was used to evaluate the performance of our method for protein remote homology detection, which is available at http://noble.gs.washington.edu/proj/svm-pairwise/. This benchmark has been used by many studies of remote homology detection methods [8], [20], [34], which can provide good comparability with previous methods. The benchmark contains 54 families and 4352 proteins selected from SCOP version 1.53. These proteins are extracted from the Astral database [47] and include no pair with a sequence similarity higher than an E-value of 10^−25^. For each family, the proteins within the family are taken as positive test samples, and the proteins outside the family but within the same superfamily are taken as positive training samples. Negative samples are selected from outside of the superfamily and are separated into training and test sets.

### Amino acid indices

The Amino Acid Index (AAIndex) [43] is a database of numerical indices representing various physicochemical and biochemical properties of amino acids and pairs of amino acids (http://www.genome.jp/aaindex/). There are three sections in the latest version of the database (version 9): AAIndex1, AAIndex2 and AAIndex3. AAIndex1 contains 544 indices; AAIndex2 has 94 amino acid mutation matrices; AAIndex3 has 47 statistical protein contact potential matrices. Because AAIndex2 and AAIndex3 are matrices, they are not suitable for the physicochemical distance transformation. Therefore, the AAIndex1 is selected for protein transformation step. After removing the incomplete data and the indices with all zeros in AAIndex1, 531 indices are selected for the physicochemical property distance transformation. All the 531 indices are available at Text S1.

### Generation of frequency profiles

In order to incorporate the evolutionary information into the prediction, the frequency profiles are calculated. A frequency profile can be represented as a matrix *M*, its dimensions are *L*×20, where *L* is the length of the protein sequence and 20 is the number of the standard amino acids. Each element of *M* is the target frequency which reflects the probability of an amino acid occurring at a specific sequence position during evolutionary processes. The target frequency is calculated from the multiple sequence alignments generated by running PSI-BLAST [48] against the NCBI's NR dataset with parameters (-j 10, -e 0.001). Only the multiple sequence alignments with sequence identity less than 98% are used for the calculation. The target frequency is calculated as:
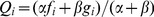
(2)where *β* is a free parameter set to a constant value of 10 which is initially used by PSI-BLAST and *α* is the number of different amino acids in a given column minus one. *g_i_* is the pseudo-count for amino acid *i*, which can be calculated as:
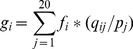
(3)where *f_i_* is the observed frequency of amino acid *i*, *p_j_* is the background frequency of amino acid *j*, *q_ij_* is the score of amino acid *i* being aligned to amino acid *j* in BLOSUM62 substitution matrix, which is the default score matrix of PSI-BLAST.

### Physicochemical distance transformation

In order to incorporate the sequence-order effects into the prediction, the physicochemical distance transformation (PDT) is proposed to represent the sequence-order information of the proteins. There are two kinds of PDTs: one is the sequence-based PDT, which only uses the protein sequence information; another one is the profile-based PDT, which uses the evolutionary information represented in the frequency profiles. The following details the process of the sequence-based PDT.

Given a protein *P* with *L* amino acids: 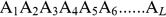
(4)


Where A_1_ is the amino acid at protein chain position 1, A_2_ is the amino acid at protein chain position 2 and so forth. Given an amino acid index *j* in AAindex1, each protein sequence is converted into a series of numbers using the amino acid index *j*.

**Figure 4:**
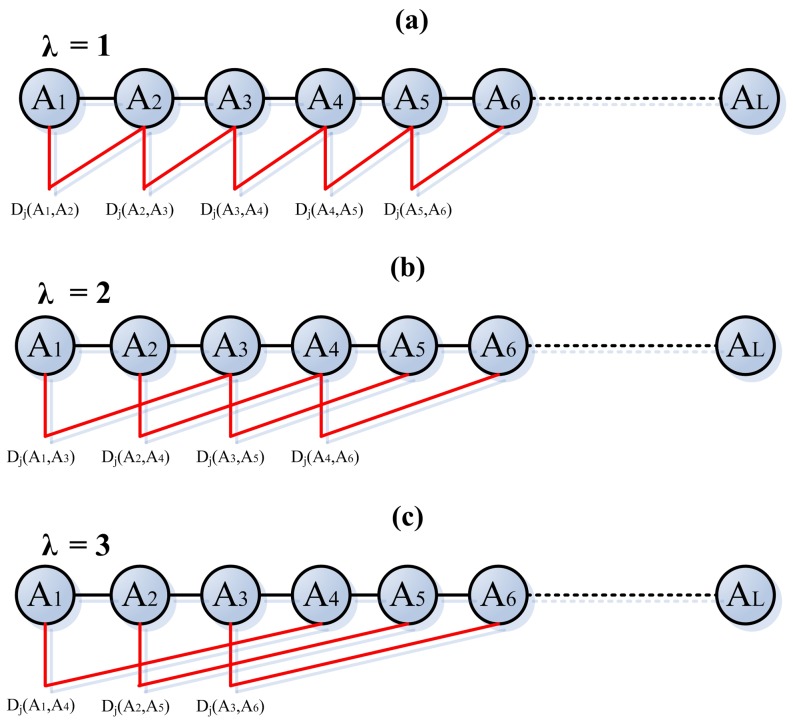
A schematic diagram of physicochemical distance transformation approach with λ values of 1 (subfigure a), 2 (subfigure b) and 3 (subfigure c). A_1_ is the first amino acid in the protein sequence; A*_L_* is the Lth amino acid in that protein. D*_j_*(A*_i_*,A*_i+λ_*) is calculated by equation (4) based on index j in AAIndex, which measures the correlation between any two amino acids with a distance λ along the protein sequence. The sequence-order information associated with the physicochemical properties can be efficiently reflected by equation (3) and (4)



The sequence-order information associated with the physicochemical properties can be efficiently reflected by the following equation (Figure 4):

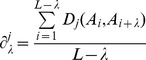
(5)


Where λ is the distance between two amino acids along the protein chain, *D_j_*(*A_i_,A_i+λ_*) can be calculated by the following equation :
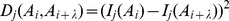
(6)


Where *I_j_*(*A_i_*) and *I_j_*(*A_i+λ_*) represent the normalized physicochemical property values of amino acid *A_i_* and *A_i+λ_* in index *j*, which are calculated by the following equation:
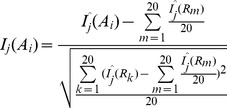
(7)


Where *I^?^*(*A_i_*) represents the raw physicochemical property value of amino acid *A_i_* in index *j*, *R_m_* (*m* = 1, 2, 3, 4, …, 20) represents the 20 standard amino acids.

In such a way, the total number of the sequence-order information variables 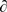 can be calculated as 531*β, where 531 is the total number of indices in the AAIndex1, *β* is the maximum of *λ* (*λ* = 1, 2, …, *β*). Each protein sequence is represented as a vector with elements of the 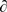 variables. Therefore, the length of the feature vector is equal to the total number of 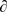 variables (531*β). The source code of the physicochemical distance transformation is available at Text S2.

**Figure 5:**
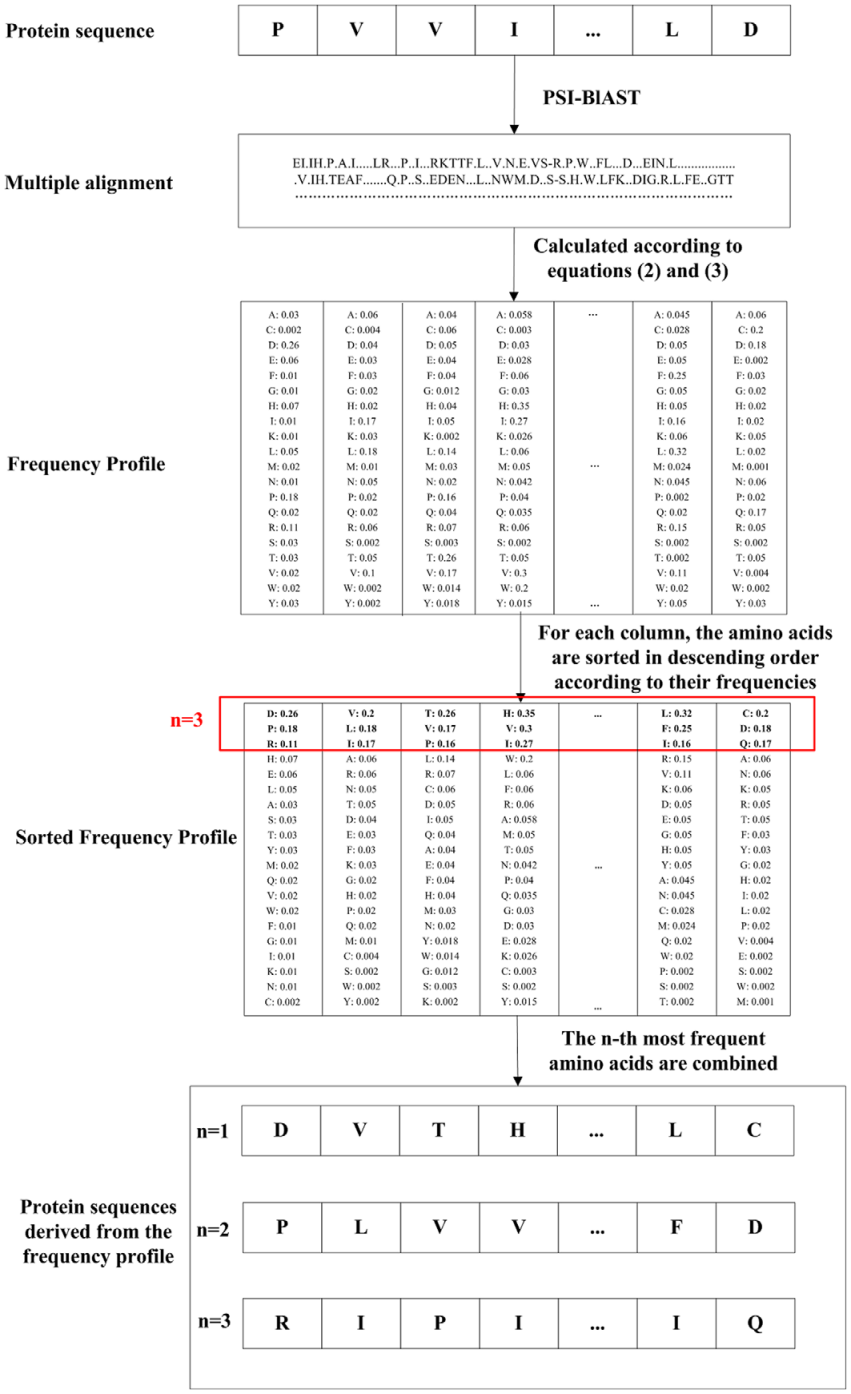
The flowchart of generating the profile-based protein sequences. The multiple sequence alignment is obtained by PSI-BLAST. The frequency profile is calculated from the multiple sequence alignment. For each column in the frequency profile, the amino acids are sorted in descending order according to their frequencies, and then the profile-based sequences are obtained by combining the n-th most frequent amino acids.

The process of profile-based PDT is similar as that of the sequence-based PDT, except that there is an additional step of extracting the evolutionary information from the frequency profiles. The target frequencies in the frequency profiles reflect the probabilities of the corresponding amino acids appearing in the specific sequence positions. The higher the frequency is, the more likely the corresponding amino acid occurs. It is reasonable to use the *n*-th most frequent amino acids in the frequency profiles to represent the protein sequences. Each amino acid in a protein sequence is replaced by its corresponding *n*-th most frequent amino acid in the frequency profile. Therefore, the resulting protein sequence takes the evolutionary information in the frequency profile into consideration. The flowchart of generating the profile-based protein sequence is shown in Figure 5. The PDT performs on the resulting protein sequence. The rest steps are the same as those of the sequence-based PDT. The length of the feature vector of the profile-based PDT is 531*β*n. In this study, the first and second most frequent amino acids are investigated, since in our previous study we demonstrated that these amino acids showed strong discriminative power [23].

### Support vector machine

Support vector machine (SVM) is a class of supervised learning algorithms first introduced by Vapnik [7]. Given a set of labelled training vectors (positive and negative input samples), SVM can learn a linear decision boundary to discriminate the two classes. The result is a linear classification rule that can be used to classify new test samples. When the samples are linearly non-separable, the kernel function can be used to map the samples to a high-order feature space in which the optimal decision boundary can be found. SVM has exhibited excellent performance in practice and has a strong theoretical foundation of statistical learning.

In this study, the publicly available Gist SVM package (http://www.chibi.ubc.ca/gist/) is employed. The SVM parameters are used by default of the Gist Package except that the kernel function is set as either a quadratic or a radial basis function.

### Evaluation methodology

Because the test sets have many more negative than positive samples, simply measuring error-rates will not give a good evaluation of performance. For the cases in which the positive and negative samples are not evenly distributed, the best way to evaluate the trade-off between the specificity and sensitivity is to use a receiver operating characteristics (ROC) score [49]. A ROC score is the normalized area under a curve that plots true positives against false positives for different classification thresholds. A score of 1 denotes perfect separation of positive samples from negative ones, whereas a score of 0 indicates that none of the sequences selected by the algorithm is positive. Another performance measure is ROC50 score, which is the area under the ROC curve up to the first 50 false positives.

## Supporting Information

Text S1 **The information of all the 531 indices used for the experiments.**
(TXT)

Text S2 **Source code of physicochemical distance transformation.**
(RAR)
